# Characterization and Biological Evaluation of Composite Nanofibrous Membranes Prepared from Hemp Salmon (*Oncorhynchus keta*) Skin Collagen

**DOI:** 10.3390/cells14070537

**Published:** 2025-04-03

**Authors:** Yu Liu, Mochi Zhu, Rui Duan, Junjie Zhang

**Affiliations:** 1 School of Marine Food and Bioengineering, Jiangsu Ocean University, Lianyungang 222005, China; 2022220843@jou.edu.cn (Y.L.);; 2 Co-Innovation Center of Jiangsu Marine Bio-Industry Technology, Jiangsu Ocean University, Lianyungang 222005, China; hy203204@163.com; 3 Jiangsu Key Laboratory of Marine Bioresources and Environment, Jiangsu Ocean University, Lianyungang 222005, China; 4 School of Marine Science and Fisheries, Jiangsu Ocean University, Lianyungang 222005, China; 5 Jiangsu Institute of Marine Resources Development, Jiangsu Ocean University, Lianyungang 222005, China

**Keywords:** collagen, electrostatic spinning, cell viability, wound healing, biomaterials

## Abstract

Aquatic collagen, a natural macromolecule protein with excellent biocompatibility, has attracted attention in the field of medical materials. Compared to mammalian collagen, aquatic collagen offers unique advantages, including the absence of zoonotic disease risks and religious concerns. In this study, salmon skin collagen nanofiber membrane (GS) was prepared by electrostatic spinning. Then, skin collagen was combined with silk sericin (SS) and sodium hyaluronate (HA) to fabricate composite collagen nanofiber membrane (GF) using electrostatic spinning technology. GF membranes were further cross-linked (GFL) for use in a mouse wound healing model. The physicochemical properties and biocompatibility of GS, GF, and GFL were evaluated. FTIR analysis revealed that GFL exhibited a more stable secondary structure compared to GS and GF. DSC and TGA results indicated that GFL had the highest thermal stability, followed by GF. Cytotoxicity tests confirmed that GS, GF, and GFL were non-cytotoxic, with GF showing the highest cell viability rate of 175.23 ± 1.77%. In the wound healing model, GFL group achieved nearly complete healing by day 14 (98 ± 0.1%), compared to 76.04 ± 0.01% in the blank group. Measurement of TGF-β1 and VEGF levels in the healing tissue on day 14 indicated that the GFL group had progressed to the late stage of healing, whereas the blank group remained in the early stage. These results suggest that GFL holds significant potential as a medical biomaterial for wound healing applications.

## 1. Introduction

**Figure 1:**
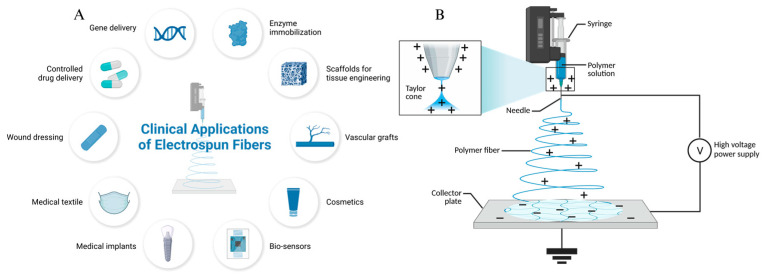
Electrostatic spinning application areas (**A**) and electrostatic spinning schematic (**B**).

Nanofibrous membranes produced through electrostatic spinning offer considerable design flexibility in terms of composition and structure, thanks to their large specific surface area and porosity [1]. These membranes present clear advantages in the field of biomaterials (Figure 1A) [2]. The electrostatic spinning process involves applying a high-voltage electric field to both ends of the polymer [3,4,5,6]. When the electrostatic force exerted on the polymer liquid exceeds the surface tension, the material is stretched into nanofibers and collected on a receiver (Figure 1B), resulting in nanofiber matrices that mimic the structure of the extracellular matrix (ECM).

Biopolymers such as collagen [3], chitosan [7], sodium hyaluronate [8], and hydroxyapatite [9] are widely utilized in wound healing dressings due to their biocompatibility and biodegradability [1]. Collagen, a primary component of the ECM [10], is a functional protein that plays a crucial role in maintaining skin elasticity, regulating human mobility and immunity, inducing cell viability and differentiation, lubricating joints, and promoting wound healing [11]. Currently, the collagen used for electrostatic spinning predominantly originates from pig skin and cowhide [12]. However, the application of such collagen in wound dressings is limited due to zoonotic risks and religious restrictions, making collagen derived from fish skin a highly effective alternative. Fish skin collagen has a lower denaturation temperature and is more readily broken down and absorbed by the body compared to mammalian collagen [13]. In recent research, dissolved fish collagen mixed with polyvinyl alcohol in a 1,1,1,3,3,3-hexafluoropropanol (HFIP)-acetic acid-DMSO solution, successfully obtaining bacteriostatic nanofibrous membranes through electrostatic spinning [14]. Electrostatic spinning cod (*Micromesistius*) poutassou skin collagen dissolved in a citric acid-HCl solution had better moisture resistance of collagen nanofibrous membranes [15]. The blended electrostatic spinning of blue shark (*Prionace glauca*) type II collagen and chitosan has resulted in nanofibrous membrane dressings with notable bacteriostatic characteristics [16]. Additionally, composite nanofibers created by electrostatic spinning marine fish collagen (CL) and sericin protein (SF) effectively support the formation of the ECM, encourage cell viability, and enhance immune function in areas requiring wound recovery [17].

Hemp salmon (*Oncorhynchus keta*) is a cold-water, anadromous migratory fish, with annual production capacity of 1 million tons [18]. About 30% of the total amount of fish skin is generated during processing [19]. Salmon skin is rich in collagen [20], which can upregulate antimicrobial peptides, promote angiogenesis, increase collagen deposition, and accelerate the wound healing process. Moreover, the antioxidant properties of fish collagen aid in scavenging free radicals and protecting cells from oxidative stress, highlighting its significant advantages in promoting wound healing, providing antioxidant effects, combating aging, enhancing cardiovascular health, and potentially extending lifespan [21]. Yunoki reported the preparation and cross-linking of salmon de-antigenized collagen (SC) fiber membranes [20]. The melting temperature of the membrane was increased from 28 °C to 47 °C, which significantly enhanced its thermal stability and mechanical strength. The membrane had better cytocompatibility than mammalian collagen.

In this study, salmon skin collagen was utilized as the raw material for the preparation of nanofiber membranes (GS), composite nanofiber membranes (GF), and cross-linked composite nanofiber membranes (GFL). These abbreviations are named according to the Chinese pronunciation. The three types of membranes were evaluated in terms of their physicochemical properties and biocompatibility to provide a reference for the exploration of aquatic collagen in the biomaterial fields.

## 2. Materials and Methods

### 2.1. Materials

Salmon skin collagen was provided by Collagen Laboratory of Jiangsu Ocean University (Lianyungang, China); silk sericin and sodium hyaluronate were purchased from Huaxi Biotechnology Co., Ltd. (Qingdao, China). NIH-3T3 cells were purchased from Kunming Wildlife Cell Bank of Chinese Academy of Sciences (Kunming, China). Trypsin-EDTA (0.25%) was purchased from Thermofisher Co., Ltd. (Shanghai, China). CCK-8 kit, mouse TGF-β ELISA kit, human VEGF ELISA kit, penicillin/streptomycin solution, DMEM medium, foetal bovine serum, phosphate buffer, and other reagents were purchased from Sangon Biotech Co., Ltd. (Shanghai, China). 1-ethyl-3-(3-dimethylaminopropyl)carbodiimide(EDC), N-hydroxysuccinimide (NHS) were purchased from Sigma Aldrich Trading Co., Ltd. (Shanghai, China).

### 2.2. Methods

#### 2.2.1. Preparation of Nanofibrous Membranes

Nanofibrous membranes were prepared using an electrostatic spinning device (DP30, Yunfan Technology Co., Ltd., Tianjin, China). The salmon skin collagen (SSC) was dissolved in 40% (*v/v*) acetic acid aqueous solution at the concentration of 10% (*w/v*). 1% (*w/v*) HA, 10% (*w/v*) SS was added and mixed well. The solution was loaded into a 5 mL syringe and pumped at a flow rate of 0.315 mL/h. The syringe had a metal needle size of 18 G with a nominal inner diameter of 0.84 mm and an outer diameter of 1.27 mm. A positive voltage of 20 KV was used, and the distance from the tip to the collector was 15 cm. The collection time was fixed at 127 min. All measurements were repeated three times at room temperature (25 ± 1 °C). The nanofibre membrane (GS) and composite nanofibre membrane (GF) obtained from the collection were placed in a desiccator for dry storage.

#### 2.2.2. Cross-Linking of Nanofibre Membranes

EDC/NHS (2:1) was added into 95% ethanol solution to formulate a 5% (*w/w*) cross-linking solution. The GF (50 mm × 50 mm) was placed in the solution for 24 h and then dried at 40 °C for 48 h to obtain the cross-linked composite nanofibre membrane (GFL).

#### 2.2.3. Scanning Electron Microscopy (SEM)

Ion sputtering apparatus (SBC12, CSCI Technology Co., Ltd., Beijing, China) was used to spray gold on the surface of GS, GF, and GFL. A cold field emission scanning electron microscope (Regulus8100, Hitachi Ltd., Tokyo, Japan) was used for morphological characterisation of the samples. Statistical analysis of the diameter distribution of the nanofibres was performed using Origin 2018 (Origin Laboratories Inc., Denver, CO, USA).

#### 2.2.4. Fourier Transform Infrared Spectroscopy (FTIR)

GS, GF, and GFL were scanned and analysed using an FTIR spectrometer (Perkin Elmer, Wellesley, MA, USA). The scanning range was 4000–400 cm^−1^ with an interval of 1 cm^−1^ and a resolution of 4 cm^−1^. The amide I bands were baseline calibrated and Gaussian deconvolution and fitted by second-order derivatives through the software Omnic V8.2 (Thermo Fisher Scientific Inc., Waltham, MA, USA)and Peak Fit v4.12 (Cabit Information Technology Co., Ltd., Shanghai, China), and the area share of each subpeak after the fitting was the share of the corresponding conformational content [22].

#### 2.2.5. Differential Scanning Calorimetry and Thermogravimetry Analysis (DSC and TGA)

A simultaneous thermal analyser (STA8000, Perkin Elmer, Wellesley, MA, USA) was used to analyse the thermal properties of GS, GF, and GFL nanofibrous membranes. An 8 mg sample was weighed into a crucible and heated from 30 °C to 400 °C at a rate of 10 °C/min in a gas environment with a nitrogen flow rate of 20 mL/min [23].

#### 2.2.6. Determination of Tensile Stress

The tensile stress of the samples was determined using the method in GB/T 1040.3-2006 [24]. The samples were made into a dumbbell shape of 50 × 5 mm and then measured by a mass tester (TMS-PRO type, FTC Corporation, Alexandria, VA, USA), which was stretched at a speed of 10 mm/min until the samples broke, and the measurement was repeated for three times to take the average value. The stress values were calculated according to the Equation:

$$\sigma \left(\mathrm{Mpa}\right)=F/A$$

in which F was the corresponding load measured and A (1.55 mm^2^) was the original cross-sectional area of the specimen.

#### 2.2.7. Cytocompatibility Evaluation

Sample cytotoxicity was evaluated by referring to Cai’s method with appropriate modifications [24]. Mouse fibroblasts (3T3 cells) were used to culture in Dulbecco’s modified eagle medium (DMEM medium) (containing 10% foetal bovine serum, 1% penicillin/streptomycin), 37 °C CO_2_ incubator (Galaxy S, RS Biotech, Borehamwood Hertfordshire, UK), and passaged every 2~3 d. A 5 mg sample (GS, GF, or GFL) was fully dissolved in DMEM medium to prepare a series of concentrations: 2.5 mg/mL, 1.25 mg/mL, 0.625 mg/mL, 0.3125 mg/mL, and 0.15625 mg/mL.GS and GF could be dissolved in DMEM medium in 1–2 min. GFL, despite its maximum cross-linking, was fully solubilized within 10 min. Therefore, GS, GF, and GFL do not gel during dissolution. The prepared solutions were sterilized using a 0.22 μm filter membrane and subsequently incubated at 37 °C in a CO_2_ incubator for 24 h.

3T3 cells were inoculated in 96-well plates at a density of 5000/well, cultured at 37 °C for 24 h, and divided into three groups: blank group (3T3 cells), negative control group (DMEM medium), and experimental group (each concentration of the extract), with six parallels per group. 100 μL was added to each group, and 10 μL of CCK-8 was added after incubation for 24 h. After incubation for 1 h, the OD value was measured at 450 nm (the blank group was in complete medium), and the cell viability rate was calculated according to the following formula:

$$Cell\;viability\;rate\left(\%\right)=\frac{\left(A-A_{0}\right)}{\left(A_{1}-A_{0}\right)}\times 100\%$$

The formula:

A—OD450 value of the sample group;

A_0_—OD450 value of the blank group;

A_1_—OD450 value of the control group.

#### 2.2.8. Wound Healing

Healthy male Kunming mice (KM mice, SPF grade, 4–5 weeks, 20~25 g) were purchased from Spivey (Beijing) Biotechnology Co., Ltd. (Beijing, China), and the experiment was started after 7 d of acclimatisation feeding. The KM mice were randomly divided into two groups, which were blank group (bandage, BK) and experimental group (GFL). Mice were restrained and injected intraperitoneally with 0.3 mL of tribromoethanol. After successful anesthesia, the hair on the back of mice was removed with an electric shaver. The skin was sterilized with iodophor, deiodinated with 75% ethanol. The dorsal skin of the mice was excised using an 8 mm diameter skin punch, creating two standardized wounds to expose the underlying musculature. The wounds were sterilized with 75% ethanol, and the limbs of the mice were abducted and fixed to the surgical plate with rubber bands. The GFLs and bandages at the wound size covered the wound surface. The mice were housed in a single cage after surgery. The performance of the mice was observed and recorded every day, including daily mental state and water and food intake, whether or not there was any restriction of activity. Wounds were photographed at 0, 7, 14, 21, and 24 d after surgery, and the healed area was analysed by software. The wound healing rate was calculated according to the formula:

Wound healing rate (WHR, %) = Healed area/Original wound area × 100%

#### 2.2.9. TGF-β and VEGF Expression Measurement

The mice were euthanized on day 14. The wound edges were marked with a marker pen, and ophthalmic scissors were used to flare out 2–3 mm along the wound edges (including the healing centre and surrounding tissue) to a depth of the fascial layer. The tissues were rinsed with pre-cooled PBS (0.01 M, pH = 7.4) to remove residual blood, weighed, and then sheared. The sheared tissues were added into a glass homogeniser with the corresponding volume of PBS and ground thoroughly on ice. The mixture was centrifuged, and the supernatant was collected. The expression levels of TGF-β_1_ and VEGF were quantified using mouse TGF-β ELISA kit and human VEGF ELISA kit (Sangon Biotech Co., Ltd., Shanghai, China).

### 2.3. Statistical Analysis

All data graphs were designed by Origin 2018 (Origin Laboratories Inc., Denver, CO, USA), and the data were statistically analyzed by IBM SPSS Statistics 20 (IBM, Amonk, NY, USA).

## 3. Results and Discussion

### 3.1. Morphological Characteristics

**Figure 2:**
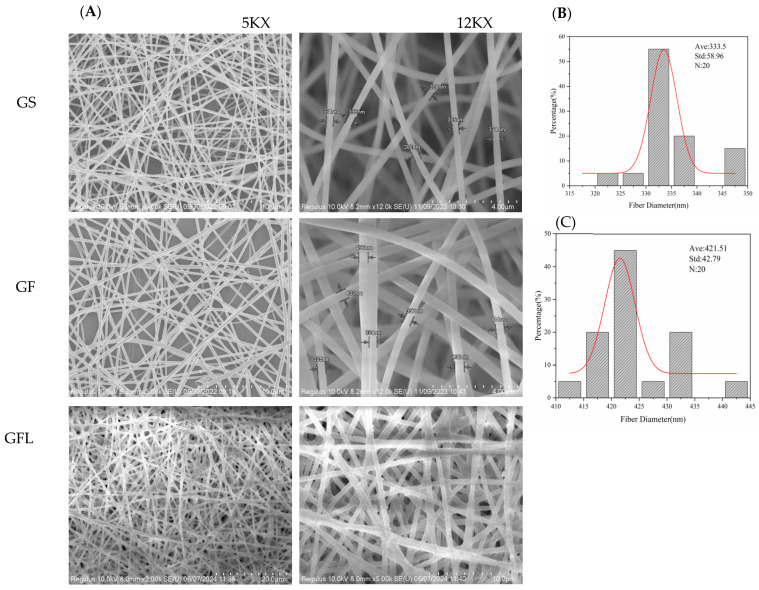
SEM results and diameter distribution statistics of GS, GF, and GFL. (**A**) GS, GF, and GFL nanofibers. (**B**) Statistical results of GS diameter distribution. (**C**) Statistical results of GF diameter distribution.

Figure 2 presents the scanning electron microscopy (SEM) images and fiber diameter distribution results for GS, GF, and GFL. As illustrated, the fiber surface of the GS nanofiber membrane is smooth and flat, exhibiting no adhesion, with uniform and neat thickness [25]. It possesses a homogeneous structure interlaced with a unique spatial arrangement, resembling the fiber morphology of GF. It indicated that the addition of HA and SS did not significantly alter the spatial configuration of the nanofibrous membrane. The commonly used cross-linking agents, such as EDC/NHS and Genipin, and had a substantial impact on the fibrous structure of collagen nanofibrous membranes, leading to fiber dissolution and curling, as observed in their SEM results [26]. In this study, the cross-linked GFL has maintained its regular fiber structure as GF. However, the surface of the GFL fibers appears rough, likely due to local aggregation or adhesion between fibers during the cross-linking process, especially during the solvent evaporation stage. The uneven adhesion between fibers may result in rough or irregular structures [27].

The fiber diameter of GF was predominantly distributed around 421.51 ± 42.79 nm, which represents an increase compared to the GS fibers, measuring 333.5 ± 58.96 nm (Figure 2B,C). The hydrogen bonding between HA and SS molecules present in GF enhances the interaction force among the molecular chains, resulting in increased viscosity [28]. The viscosity of the electrostatic spinning solution significantly influences the uniformity of the continuous fibers and their diameter; higher viscosity correlates with larger nanofiber diameters [29].

### 3.2. Fourier Transform Infrared Spectroscopy (FTIR)

**Figure 3:**
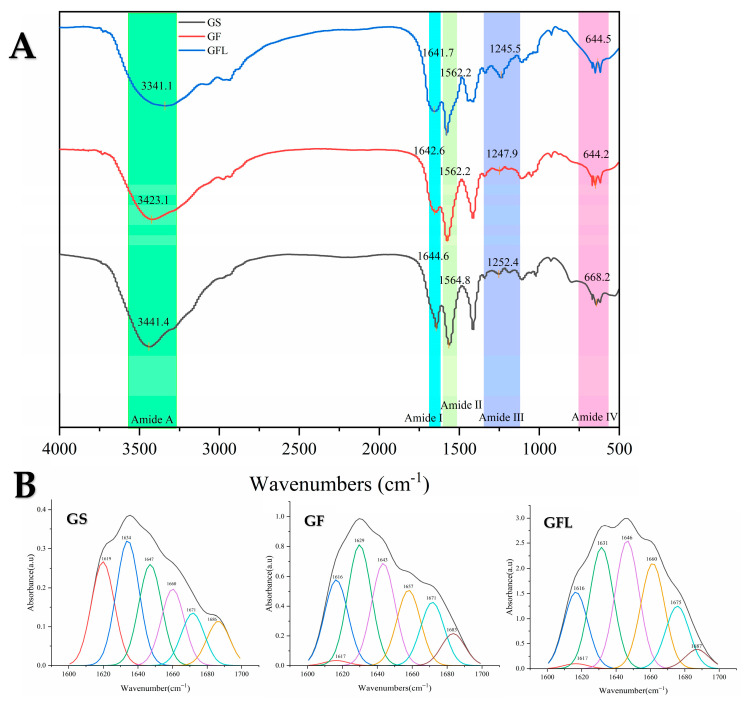
FTIR scan spectra (**A**) and protein secondary structure fitting diagram (**B**) of GS, GF, and GFL.

Figure 3A displays the FTIR spectra of GS, GF, and GFL. The primary characteristic bands of GS include the amide A band at 3441.4 cm^−1^, the amide I band at 1644.6 cm^−1^, the amide II band at 1564.8 cm^−1^, the amide III band at 1252.4 cm^−1^, and the amide IV band at 668.2 cm^−1^ [30,31]. In comparison to GS, the amide A band of GF shifts to 3423.1 cm^−1^. The amide and hydroxyl groups in collagen can generate hydrogen bonds with the hydroxyl and carboxyl groups of HA, as well as with the hydroxyl and amino groups of SS. The resultant increase in hydrogen bonding strengthens the hydrogen bond interactions, leading to a decrease in the vibrational frequency of the N-H group, which accounts for the rightward shift of the amide A band [32]. The amide I band of GF is observed at 1642.6 cm^−1^, signifying a transformation in part of the protein’s secondary structure, which results in a more compact molecular arrangement. This compactness reduces the vibrational freedom of the carbonyl group (C=O), causing the absorption peaks to shift toward lower wavenumbers. Additionally, the enhanced hydrogen bonding leads to leftward shifts in the amide II, amide III, and amide IV bands of GF. This finding is corroborated by the study conducted by Zhou et al., which states that the interaction (generation of hydrogen bonds) between filaggrin and collagen does not impact the protein’s secondary structure [33]. The positions of the amide I–IV; bands of collagen exhibit minimal shift after spinning with silk sericin. The increase in hydrogen bonding within GF enhanced its thermal stability and mechanical properties.

Following cross-linking using EDC-NHS, the amide A band of GFL is noted to shift right to 3341.1 cm^−1^. EDC-NHS facilitates the formation of covalent amide bonds between the carboxyl and amino groups within the collagen molecule through activation [34]. This covalent cross-linking not only fortifies the intermolecular connections but may also lead to a compression of the molecular structure, thereby reducing the distance between the N-H group and adjacent groups, which promotes an enhanced hydrogen bonding network [35]. Consequently, the frequency of N-H stretching vibrations diminishes, resulting in the rightward shift of the amide A band toward lower wavenumbers [36]. The amide I, amide II, amide III, and amide IV bands of GFL exhibit slight leftward shifts, suggesting that the increased hydrogen bonding correlates with an elevation in the frequencies of the N-H bending vibration, the C-N stretching vibration, and the vibration frequency of the carbonyl group (C=O).

The amide A and I–III bands represent key characteristic peaks that confirm the backbone structure of the protein peptide chain. Notably, the amide I band (1600–1700 cm^−1^) reflects the cumulative contributions of various protein secondary structure conformations [37]. The amide I bands for GS, GF, and GFL were analyzed and fitted using Omnic and Peakfit software, with secondary structure ratios calculated based on the areas associated with each structural conformation (Table 1).

As illustrated in Table 1, GS exhibits a relatively balanced distribution of secondary structures, with *β*-sheet and *β*-turn content at 24.79% and 28.70%, respectively. This suggests a stable protein structure that preserves overall integrity through the flexible connections provided by *β*-sheets. Furthermore, the presence of appropriate levels of random coil and *α*-helix indicates potential functional adaptability, enabling the protein to engage in various biological activities through structural modifications [38].

In contrast, GF displays a higher *β*-sheet content than GS, attributable to the extensive hydrogen bonding interactions between collagen, hyaluronic acid (HA), and silk sericin (SS) [32]. These interactions are vital for the formation and stabilization of *β*-sheets [39]. The increased hydrogen bonding facilitates tighter connections among adjacent polypeptide chains through the bonding of carbonyl and amino groups, thus enhancing the stability of the *β*-sheet structure [40]. Additionally, GF partially converts α-helical regions into *β*-sheets. Given that silk sericin inherently favors *β*-sheet formation [41], the overall redistribution of hydrogen bonds during mixing diminishes the α-helix content and enhances the *β*-sheet formation in GF [42]. The presence of *β*-turns imparts structural flexibility to the proteins, allowing for bending and folding. However, the observed reduction in *β*-turn content in GF indicates a decrease in structural flexibility in favor of a more organized (*β*-sheet) configuration, which suggests a more compact and stable structure for GF [43].

The GFL exhibits a secondary structure composition of 47.16% *β*-sheet, 22.64% irregular coiling, 14.38% α-helix, and 15.81% *β*-turn. *β*-turn and *β*-sheet content of GFL increase, whereas the α-helix content decreases. The activation of the carboxyl (-COOH) group by EDC facilitates its reaction with the amino group (-NH2) to form stable amide bonds. In contrast, the α-helix formation and stabilization rely significantly on intra-chain hydrogen bonding, whereas the *β*-sheet structure is dependent on hydrogen bonding between molecular chains [44]. Cross-linking enhances intermolecular interactions, and the establishment of a network of intermolecular chain hydrogen bonds may promote the transformation of the α-helix into a more stable *β*-sheet structure [45]. This process allows for the binding of collagen and SS amino groups to the carboxyl groups of HA via cross-linking agents, thereby creating a more robust three-dimensional network structure.

### 3.3. Differential Scanning Calorimetry and Thermogravimetry Analysis (DSC and TGA)

**Figure 4:**
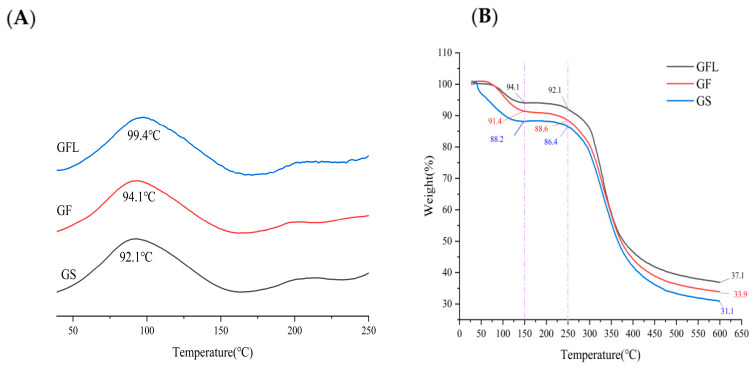
DSC (**A**) and TGA (**B**) weight loss curves of GS, GF, and GFL.

Figure 4A illustrates that GS, GF, and GFL each exhibit an absorption peak. A higher melting temperature (Tm) value indicates improved thermal stability, as dehydration of collagen at this temperature, along with the breaking of hydrogen bonds, leads to the disintegration of the collagen tetra- and tertiary structures, resulting in a transition from the α-helix and *β*-sheet structures to a randomly coiled configuration [46,47]. The thermal stability of GF is slightly superior to that of GS, whereas GFL demonstrates the highest thermal stability. The incorporation of HA and SS in GF increases partial hydrogen bonding compared to GS, thereby enhancing thermal stability [48]. Furthermore, the EDC-NHS cross-linked GFL establishes covalent linkages between carboxyl and amine groups in the proteins through the formation of amide bonds. This covalent bonding significantly improves the inter- and intramolecular structural stability of the protein, making it more resistant to decomposition at elevated temperatures. Moreover, the Tm value of GFL is markedly higher than that of spirulina protein concentrate-collagen nanofibers prepared by Vahid et al. (74.41C) [49].

Figure 4B presents TGA results for GS, GF, and GFL. The thermal degradation process is characterized by two distinct stages: the initial stage involves the depolymerization of the macromolecular network, whereas the subsequent stage addresses further degradation following the disruption of instermolecular covalent bonds [50]. As illustrated in Figure 4B, within the temperature range of 50 °C to 150 °C, all three sample groups exhibited rapid mass loss due to the depolymerization of the macromolecular network. In the range of 150 °C to 250 °C, the mass loss occurred at a slower rate, indicative of changes in the spatial structure of the molecules, along with the dissociation of intramolecular crystalline water due to heat absorption [51].

The TGA results demonstrated that the residual weights of GS, GF, and GFL at 150 °C were 88.2%, 91.4%, and 94.1%, respectively, and at 250 °C, they were 86.4%, 88.6%, and 92.1%. The degradation onset temperature (Tonset) and maximum degradation temperature (Tmax) of the three samples were shown in Table 2, which indicated Tonset and Tmax GFL are higher than those of GF and GS. Therefore, GFL possessed superior thermal properties compared to GF. EDC reacted with carboxyl group of the aspartic and glutamic acid residue existing in the collagen matrix to form an activator, viz. the unstable derivative of urea. The use of NHS can improve the cross-linking yield of carbodiimides by forming a more stable ester [52]. On the other hand, a large number of hydrogen bonds with were formed among HA and SS collagen. It was ester and hydrogen bond that made the thermal stability of GFL higher than GF [53,54,55]. Mills [56] studied the thermal denaturation behavior of soy protein (glycinin) and found that the β-folded structure was retained even when heated to 95 °C, while the α-helix content was reduced, suggesting that the β-sheet may be related to the heat resistance of the protein. Therefore, the higher thermal stability of GFL may be correlated with its higher β-sheet content. This finding was in accordance with DSC results.

### 3.4. Determination of Tensile Stress

Table 3 showed that the tensile stress of GS and GF was 1.72 ± 0.39 MPa and 2.4 ± 0.62 MPa, respectively, indicating that the hydrogen and covalent bonds formed between collagen, HA, and SS in GF enhanced its tensile properties. GFL had the highest tensile stress of 4 ± 0.62 MPa, twice that of the GF. This is consistent with the previous reports that EDC/NHS cross-linking stabilizes collagen fibers and improves the durability and mechanical properties of the materials [49].

### 3.5. Cytocompatibility Evaluation

**Figure 5:**
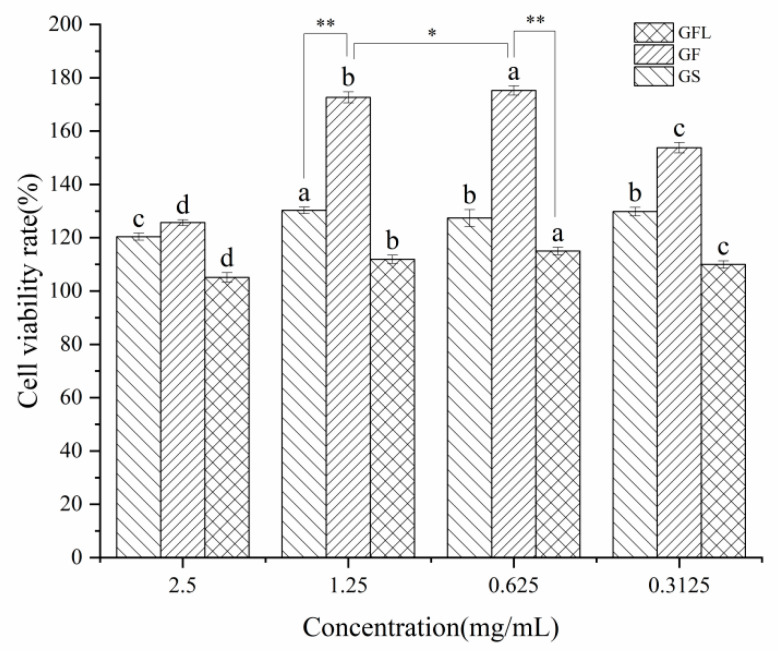
Effect of different concentrations of GS, GF, and GFL solution on cell viability. a, b, c, and d indicate significant differences (*p* < 0.05) for the same samples at different concentrations. * *p* < 0.05 indicated the difference between the two groups. ** *p* < 0.01 indicated that the difference between the two groups was extremely significant.

Figure 5 demonstrates that GS, GF, and GFL exhibited no cytotoxic effects and displayed the highest cell viability rates of 127.39 ± 3.16%, 175.23 ± 1.77%, and 115 ± 1.39%, respectively. The combination of arginine/glutamine, as well as valine, isoleucine, leucine, and hydroxyproline in collagen, contributed to a significant promotion of cell viability [57,58]. Notably, compared to GS, the cell viability rate of GF increased by 48%. This enhancement can primarily be attributed to the synergistic effect of SS protein and HA [59]. The crystalline region in SS protein is primarily composed of alanine repetitive sequences and contains Arg-Gly-Asp sequences, which serve as biorecognition signals to facilitate cell adhesion and viability [60,61,62,63,64]. It has been reported that incorporating an appropriate quantity of SS as a substitute for fetal bovine serum in serum-free culture medium during specific stages of the cell cycle can stimulate the growth of rat islet cells [65]. Yao reported the highest viability rate of 130% of L929 cells by electrostatically spun nanofiber membrane prepared from gelatin/keratin and incubated for 3 weeks. This gap with GF may be attributed to SS and HA, which showed excellent proliferative effect on the cells [66].

In contrast, the cell viability rate of GFL decreased by 60% compared to GF. This reduction can be attributed to the stronger intermolecular forces present in the cross-linked GFL, which prevented it from fully decomposing into amino acids and short peptides in the DMEM culture medium. Besides, covalent cross-linking using EDC-NHS may impact the conformation of collagen, potentially masking or interfering with the vital RGD and GFOGER sequences [67,68]. The lower cell viability rate of GFL may also be due to the remaining EDC and NHS in the material. Consequently, GFL exerted a limited effect on promoting fibroblast viability and adhesion [69]. This phenomenon aligns with findings reported by Luo, who observed that the viability rate of MC3T3 cells in EDC-NHS cross-linked collagen nanofibrous membranes was lower than that in uncross-linked collagen nanofibrous membranes [26].

### 3.6. Wound Healing

**Figure 6:**
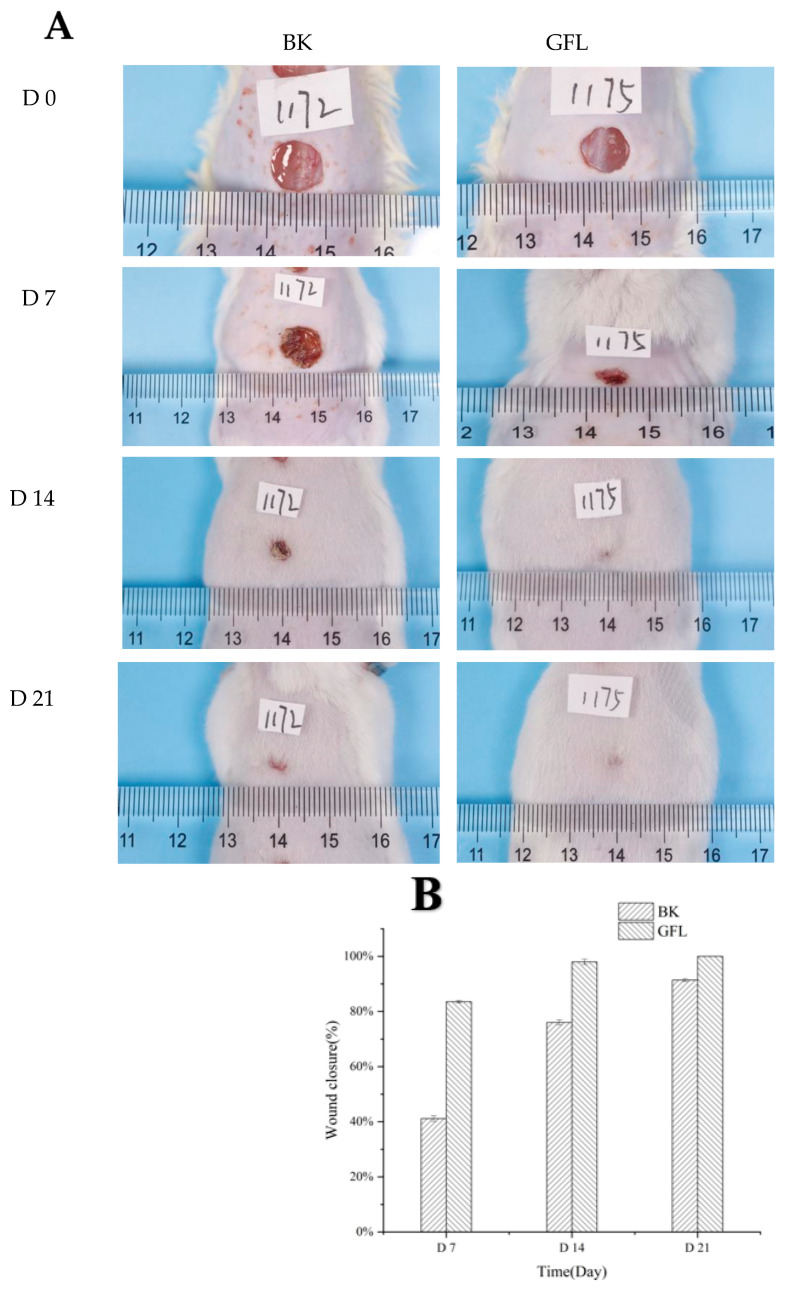

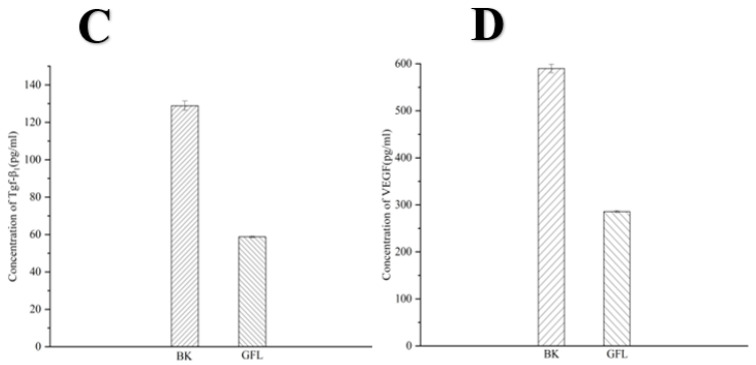
Wound healing photographs (**A**) and healing area (**B**) of GFL. Tgf-β_1_ (**C**) and VGEF (**D**) expression in the GFL model at 14 days.

GF could dissolve instantly in contact with water. When it is used as a medical dressing, GF will disintegrate quickly and cannot have a covering and protective effect on the wound. Therefore, we cross-linked the GF, hoping it could disintegrate slowly and continuously protect the wound. Healthy male Kunming mice were utilized as experimental subjects, and deep wounds with a diameter of 8 mm were created on their backs, followed by the application of GFL membranes. Throughout the wound healing period, all groups of KM mice exhibited no abnormalities in food intake, water intake, activity, or mental status, and no adverse reactions, such as redness, swelling, or allergy, were observed. GFL forms a transparent film-like consistency within 5 min when covered with wounds and slowly dissolves within 2 h. As illustrated in Figure 6A,B, a significant difference in wound healing was noted on day 7, with the blank group showing a healing rate of 41.1 ± 1.21% compared to 83.56 ± 0.69% in the GFL group. These results are in line with Zhang’s findings, which reported a mouse wound model using silk protein/collagen nanofibers as a dressing, where the healed area of the nanofibers reached 50% at day 7, compared to only 20% in the blank group [70]. At this stage, collagen is degraded into small molecular peptides that participate in cell viability and signal transduction, inducing the expression of growth factors such as VEGF and TGF-β1. By day 14, the wound healing rate in the GFL group reached 98 ± 0.1%, in contrast to 76.04 ± 0.01% in the BK group. The fourteenth day post-trauma is considered a critical phase for granulation regeneration. It is widely accepted that mature granulation tissue promotes re-epithelialization and facilitates initial wound healing, with macrophages playing a pivotal role [71]. SS and HA promote macrophage production and viability during the first 14 days of wound healing, with a decline to a steady state thereafter, which prevents macrophages from adversely affecting the formation of new blood vessels and collagen [72,73]. On day 21, 10% of the wounds in the BK group remained unhealed, whereas the wounds in the GFL group had completely healed. As shown in Figure 6C, the TGF-β1 expression in the BK group on day 14 was 128.93 ± 2.49 pg/mL, compared to 58.75 ± 0.47 pg/mL in the GFL group. This suggests that the wound healing in the GFL group had advanced to a later stage, corroborating findings from Hassani, who reported that TGF-β1 expression initially increased (0–7 days) and then decreased (day 7–14) during wound healing, showing a negative correlation with the wound healing area [71].

Figure 6D indicates that on day 14, the expression levels of VEGF were 589.91 ± 8.82 pg/mL in the BK group and 285.58 ± 2.52 pg/mL in the GFL group, both of which gradually declined as healing progressed, eventually returning to normal levels after complete re-epithelialization. At this stage, VEGF expression was primarily localized in keratinocytes and macrophages, suggesting its regulatory role in the later stages of wound healing is primarily to maintain tissue stability [74]. This observation further supports the conclusion that the GFL group had transitioned to the late stage of wound healing.

Wound healing experiments conducted in mice demonstrated that GFL positively influences the healing process. Its favorable mechanical properties and thermal stability enable it to disintegrate slowly, whereas covering the wound, maintaining moisture, and inhibiting bacterial growth. During the initial stage of wound healing (days 0–14), collagen facilitates the formation of anti-inflammatory, pro-angiogenic macrophages via the microRNA signaling pathway, whereas the c-prepeptide fragment of collagen recruits endothelial cells to promote vascularization [75]. Additionally, SS enhances the expression of fibronectin and vascular endothelial growth factor (VEGF) by modulating the NF-κB signaling pathway in the wound, thus accelerating tissue repair [76]. HA plays a crucial role in modulating the inflammatory response and reducing the transformation of fibroblasts into myofibroblasts [77], which promotes scar-free healing. In the later phase of wound healing (days 14–21), collagen binds to α2β1 integrins, activating focal adhesion kinase (FAK) and its downstream signaling pathways. This interaction results in the downregulation of TGF-β receptors, reducing cellular sensitivity to TGF-β and inhibiting TGF-β signaling [78]. Furthermore, this mechanism restricts VEGF receptor 2 (VEGFR-2) signaling by modulating SHP2 tyrosine phosphatase activity, which leads to the dephosphorylation of VEGFR-2, consequently inhibiting VEGF-mediated angiogenic signaling and preventing excessive wound hyperplasia [79].

## 4. Conclusions

This study involved the preparation of nanofibrous membranes GS, GF, and GFL through the electrostatic spinning of salmon skin collagen. FTIR spectroscopy confirmed that collagen established hydrogen bonds with HA and SS, ensuring a stable structure. The results from DSC and TGA indicated that GFL exhibited higher thermal stability compared to GF and GS, which was consistent with the FTIR findings. Both GS, GF, and GFL demonstrated pro-proliferative effects on cells. The viability rate increased by 1.75 times, at a GF solution concentration of 0.625 mg/mL. In the mouse wound healing experiment, the healing rate for the GFL group was 2.02 times faster than that of the control group (Day7) and achieved complete healing by day 14, whereas also inhibiting the expression of TGF-β1 and VEGF in the later healing stage, which minimized tissue hyperplasia and scar formation. Consequently, the GFL nanofibrous membrane demonstrates excellent physicochemical properties and high biocompatibility, making it a promising candidate for an advanced wound healing dressing.

## Figures and Tables

**Table 1:** Percentage of GS, GF, and GFL secondary structures.

	α-Helix (1650–1660)	*β*-Turn (1660–1700)	*β*-Sheet (1600–1640)	Random Coil (1640–1650)
GS	23.34%	28.70%	24.79%	23.17%
GF	15.58%	19.69%	43.63%	21.10%
GFL	14.38%	15.81%	47.16%	22.64%

**Table 2:** The degradation onset temperature (Tonset) and maximum degradation temperature (Tmax) of the GS, GF, and GFL.

Sample	Degradation Onset Temperature (Tonset)	Maximum Degradation Temperature (Tmax)
GS	38 ± 0.22 °C	324 ± 0.68 °C
GF	59 ± 0.21 °C	327 ± 0.31 °C
GFL	63.31 ± 0.51 °C	331.99 ± 0.14 °C

**Table 3:** Measurement results of mechanical properties of GS, GF, and GFL.

**Sample**	**GS**	**GF**	**GSL**
TS (MPa)	1.72 ± 0.39	2.4 ± 0.62	4 ± 0.62

## Data Availability

The data that support the findings of this study are available from the corresponding author upon reasonable request.
